# Mitochondrial neurogastrointestinal encephalomyopathy in china: a novel *TYMP* variant and comprehensive clinical-genetic insights

**DOI:** 10.1186/s13023-025-03962-3

**Published:** 2025-08-18

**Authors:** Xuebi Xu, Junhui Xia, Fei Xu, Mingshan Wang, Lihong Yang, Xiaoli Chen

**Affiliations:** 1 Department of Neurology, The First Affiliated Hospital of Wenzhou Medical University, Shangcai Village, Ouhai District, Wenzhou, Zhejiang China; 2 Department of Clinical Laboratory, Key Laboratory of Clinical Laboratory Diagnosis and Translational Research of Zhejiang Province, the First Affiliated Hospital of Wenzhou Medical University, Wenzhou, Zhejiang China

**Keywords:** MNGIE, *TYMP*, TP, ACMG, Compound heterozygous

## Abstract

**Background:**

Mitochondrial neurogastrointestinal encephalopathy (MNGIE) is a rare autosomal recessive disorder caused by variants in the *TYMP* gene, which encodes thymidine phosphorylase (TP). It is characterized by multisystem involvement, with prominent gastrointestinal, neurological, and systemic manifestations that typically exhibit progressive worsening over time.

**Methods:**

We characterized a multigenerational MNGIE family through comprehensive proband analysis, identifying compound heterozygous TYMP variants (c.131G > C, p.Arg44Pro and c.1268T>G, p.Leu423Arg in trans) as the molecular basis of disease. Extended family testing for genetic counseling confirmed no secondary pathogenic variants. Muscle biopsies were analyzed using comprehensive staining techniques. Genomic analysis involved next-generation sequencing (NGS) of the proband’s DNA and Sanger sequencing of family members’ DNA to confirm variants. In silico analysis utilized bioinformatics tools and protein modeling to predict pathogenicity and assess structural impacts, with variant classification adhering to American College of Medical Genetics and Genomics(ACMG) guidelines. Additionally, a literature review of Chinese MNGIE cases was conducted to contextualize the findings.

**Results:**

The proband exhibited characteristic MNGIE features, including gastrointestinal dysmotility, diffuse leukoencephalopathy on brain MRI (magnetic resonance imaging), and electrophysiologically confirmed peripheral neuropathy. Muscle biopsy revealed ragged red fibers, cytochrome c oxidase-deficient fibers, and enhanced succinate dehydrogenase activity in blood vessels, consistent with mitochondrial dysfunction. Genetic analysis identified a novel *TYMP* variant (c.1268T > G, p.Leu423Arg) and a known variant (c.131G > C, p.Arg44Pro) in the proband, both classified as likely pathogenic according to ACMG guidelines. Molecular analysis of other 11 family members detected heterozygous carriers of either the c.1268T > G or c.131G > C variant in six asymptomatic individuals. In silico analysis confirmed that both variants are highly conserved and likely pathogenic. Protein modeling revealed that both variants compromise structural integrity and conformation, impairing TP function. Homozygous or compound heterozygous missense variants were identified as the predominant genetic alterations in 16 Chinese MNGIE cases, with gastrointestinal and neurological symptoms being the most common clinical manifestations.

**Conclusions:**

This study enriches the variant spectrum in Chinese patients, highlights the importance of early diagnosis prior to the onset of cachexia and irreversible tissue damage, and enhances the understanding of genetic heterogeneity.

## Introduction

Mitochondrial neurogastrointestinal encephalomyopathy (MNGIE) is an ultra-rare autosomal recessive disorder first described by Okamura in 1976 [1]. It typically manifests in adolescence or early adulthood, with prominent gastrointestinal complications and neurological deficits. These include gastrointestinal dysmotility, cachexia, ophthalmoplegia, ptosis, peripheral neuropathy, and leukoencephalopathy [2]. The condition progresses relentlessly, often resulting in severe disability and reduced life expectancy.

Diagnosis relies on biochemical evidence of elevated thymidine and deoxyuridine levels in both plasma and urine, genetic confirmation of *TYMP* variants, and, in some cases, histopathological findings of mitochondrial DNA (mtDNA) abnormalities in muscle biopsies. The *TYMP* gene, located on chromosome 22q13.33, encodes TP [3]. Deficiency of this enzyme leads to systemic accumulation of thymidine and deoxyuridine [4], causing mitochondrial dysfunction through progressive secondary mtDNA variants and depletion [5]. According to the Human Gene Mutation Database(HGMD) 118 variants have been identified, spanning exonic and intronic regions, classified as variant of uncertain significance(VUS), likely pathogenic, or pathogenic [6]. Current therapeutic approaches include enzyme replacement therapy (ERT) [7]and allogeneic hematopoietic stem cell transplantation (AHSCT) [8], which aim to restore TP activity and reduce the accumulation of toxic nucleosides. Emerging strategies, including gene therapy [9–14] and substrate reduction therapy [15], are under active investigation and hold promise for addressing the underlying molecular defects.

In this study, we conducted family history investigations, clinical evaluations, neuroimaging, and genetic testing on an MNGIE-affected family. We compiled and summarized the clinical features of all documented Chinese MNGIE cases, providing a comprehensive overview of the disease’s manifestations in this population.

## Materials and methods

### Participants

**Fig. 1:**
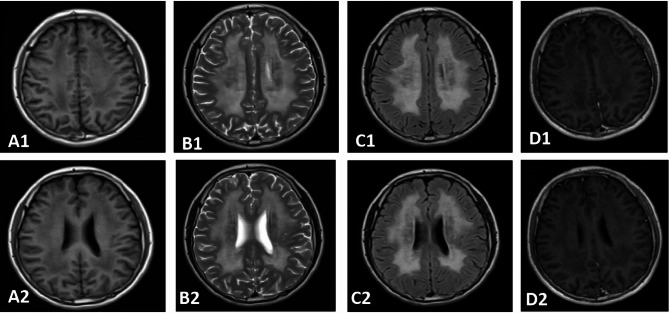
Brain MRI findings. **A1**,**A2**:T1-weighted head MRI; **B1**,**B2**:T2-weighted head MRI; **C1**,**C2**:T2flair head MRI; **D1**,**D2**: Enhanced MRI

**Fig. 2:**
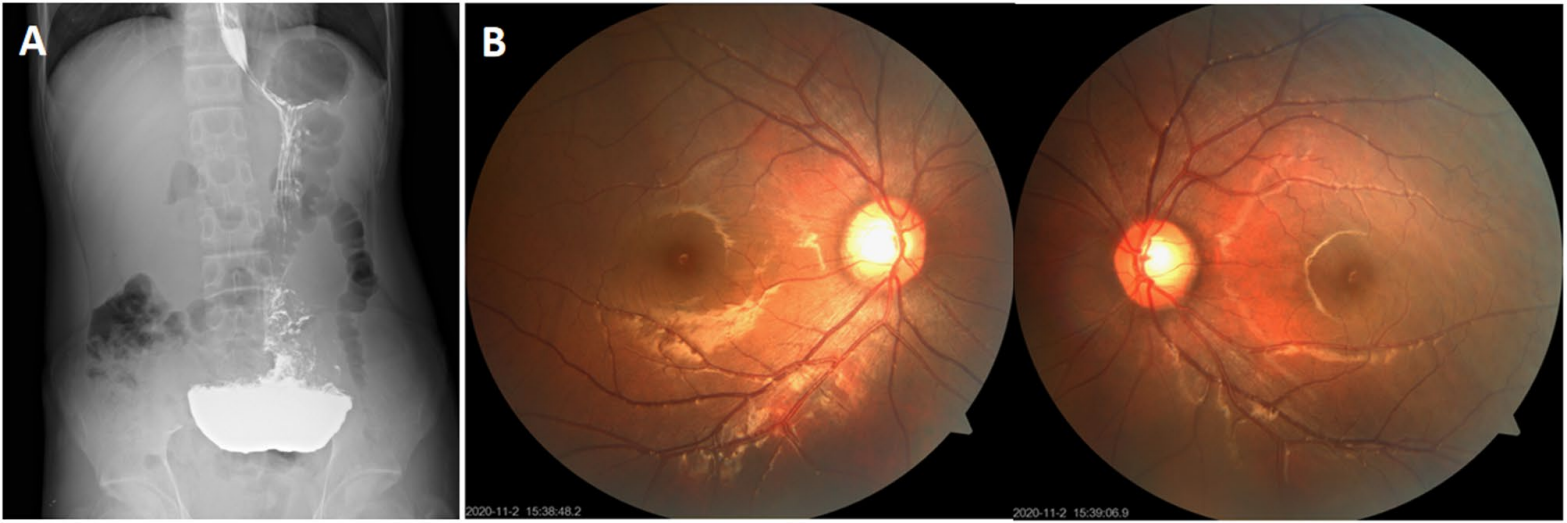
The upper gastrointestinal contrast study indicated gastroptosis (**A**). Bilateral fundus photography revealed no significant abnormalities (**B**)

The proband, a 17-year-old male, had a history of recurrent gastric distension, nausea, and vomiting since childhood. Physical examination revealed a normal height of 170 cm but significant underweight (40 kg, BMI: 13.84 kg/m²). Neurologically, he initially experienced pain in both lower extremities, followed by limb numbness and weakness after one week. Upon admission, the patient appeared emaciated and exhibited multiple neurological abnormalities, including limited right eye abduction (ophthalmoplegia), left-ear hearing loss, reduced superficial sensation in both lower limbs, and absent deep tendon reflexes in the bilateral lower limbs. Motor examination revealed weakness in the upper limbs (grade 3), mild proximal lower-limb weakness (grade 4), and severe distal lower-limb weakness (grade 0), with no pathological reflexes observed. Brain MRI demonstrated diffuse hyperintensity in the cerebral white matter on T2-weighted and fluid-attenuated inversion recovery (FLAIR) sequences (Fig. 1). Electrophysiological examinations revealed sensory and motor polyneuropathy in both the upper and lower extremities. An upper gastrointestinal barium-contrast study identified gastroptosis (Fig. 2A), while bilateral fundus photography revealed no significant abnormalities (Fig. 2B). Laboratory test showed elevated serum lactate levels (3.3 mmol/L, normal range: 0.5–2.2 mmol/L) and increased serum creatine kinase (1019 U/L, normal range: 38-174U/L). The proband’s clinical presentation, neuroradiological findings, and electrophysiological results suggested MNGIE, which was subsequently confirmed by genetic analysis. Following 4 weeks of mitochondrial-supportive nutrient therapy (including coenzyme Q10 200 mg/day, idebenone 90 mg/day, lipoic acid 600 mg/day, levocarnitine 2 g/day, and vitamin B complex daily), the patient exhibited symptomatic improvement, regaining independent ambulation, though persistent limitations in stair climbing remained. Muscle strength improved with proximal lower limbs advancing from grade 4 to 5, distal upper limbs from grade 3 to 4, and distal lower limbs achieving grade 3 from initial paralysis (grade 0). The study included 12 individuals spanning three generations, with no consanguinity identified in the pedigree analysis.

### Muscle biopsy

After obtaining written informed consent from the proband, a left biceps muscle biopsy was performed under local anesthesia. The muscle biopsy specimens were snap-frozen in liquid nitrogen-cooled isopentane and immediately cryosectioned for subsequent analysis, including the following staining procedures: hematoxylin and eosin (H&E); modified Gomori trichromatic (MGT); cytochrome c oxidase (COX); reduced nicotinamide adenine dinucleotide tetrazolium reductase (NADH); succinate dehydrogenase (SDH); and adenosine triphosphatase (ATP); periodic acid-Schiff (PAS); and oil red-O (ORO). The PAS staining was utilized for the identification of glycogen and neutral mucopolysaccharides in tissue specimens, whereas ORO staining was principally applied for the visualization of intracellular lipid accumulation.

### Genomic analysis

Venous blood samples (5 mL) were collected from the proband and other available family members into purple-top EDTA tubes using standard venipuncture. Blood samples were processed within 24 h. Total DNA was extracted following standard protocols, and gene sequencing was conducted. The proband’s DNA was selectively amplified using proprietary primers and analyzed by NGS technology at RunningGene Inc. The sequencing results were compared with the *TYMP* gene reference sequence (NCBI) using Chromas software to identify variant sites. Sanger sequencing was subsequently performed on DNA samples from other family members Finally, missense variants were confirmed through reverse sequencing.

### TP enzyme analysis

For the enzymatic assay, leukocyte layers were initially suspended in lysis buffer (50 mM Tris-HCl, pH 7.2, containing 1% Triton X-100, 2 mM phenylmethylsulfonyl fluoride, and 0.02% 2-mercaptoethanol) and subjected to brief sonication, followed by centrifugation at 20,000×g for 30 min at 4℃ to collect the supernatant for subsequent analysis. Protein concentration in the supernatant was quantified using the bicinchoninic acid (BCA) method as previously described. The enzymatic reaction was carried out in a 0.1 mL system containing 100–250 µg of supernatant protein, 0.1 M Tris-arsenate buffer (pH 6.5), and 10 mM thymidine, with incubation at 37℃ for 1 h before termination by adding 1 mL of 0.3 M NaOH. Absorbance at 300 nm was measured to determine thymine production based on the differential molar extinction coefficient (3.4 × 10³L·mol^− 1^·cm^− 1^) between thymidine and thymine under alkaline conditions, with enzyme activity expressed as nmol thymine·h^− 1^·(mg protein)^−1^. All experiments were performed in duplicate to ensure reproducibility.

**Plasma pyrimidine analysis**.

Plasma concentrations of thymidine and deoxyuridine were measured in our patient pre- and post-treatment. All those metobolites were detected by MetWare (http://www.metware.cn/) based on the AB Sciex QTRAP 6500 LC-MS/MS platform.

### In Silico analysis

The variant sites were aligned with the *TYMP* gene amino acid sequences of seven other homologous species, including humans, Chinchilla lanigera, Pan troglodytes, Callithrix jacchus, Lepus europaeus, Caviaporcellus, Elephantulusedwardi and Macacafascicularis, using the Clustal X-2.1-win software. Sequences were obtained from the NCBI database (https://www.ncbi.nlm.nih.gov/guide/homology/). The pathogenicity of the variant site was predicted using the following bioinformatics tools: Mutation Taster(https://www.mutationtaster.org/), SIFT(https://sift.bii.a-star.edu.sg/), REVEL(https://genome.ucsc.edu/cgi-bin/hgTrackUi?db=hg19&g=revel), PROVEAN(https://provean.jcvi.org/index.php), and CADD (https://cadd.gs.washington.edu/). Using the protein structure (PDB ID: P19971) obtained from UniProt (https://www.uniprot.org/) as a reference, we utilized PyMOLWin-2.3 software to analyze the spatial structural changes of amino acids before and after the variant. Subsequently, we examined the alterations in intermolecular forces to assess the functional impact of the variant on the protein.

### ACMG variant classification

The variant sites were classified according to the ACMG guidelines.

### Literature review of Chinese MNGIE cases

A comprehensive literature review was performed to identify reported cases of MNGIE in China. Systematic searches were conducted in the China National Knowledge Infrastructure (CNKI, https://www.cnki.net/),and Wanfang(https://www.wanfangdata.com.cn/) databases for Chinese-language publications, as well as in PubMed(https://pubmed.ncbi.nlm.nih.gov/) for English-language literature. The search strategy utilized the following keywords and their combinations: “MNGIE”, “mitochondrial neurogastrointestinal encephalopathy”, “Chinese”, “China”, “*TYMP* variants” and “*TYMP* mutations”. Articles were initially screened for relevance based on title and abstract, followed by full-text reviews of potentially eligible studies. Inclusion criteria required confirmed MNGIE cases with documented clinical, biochemical, and/or genetic evidence in patients of Chinese origin. Sixteen cases [16–30], including our case, meeting the inclusion criteria were analyzed, with data extraction covering demographics, clinical manifestations, genetic variants, and diagnostic findings.

## Results

### Muscle biopsy

**Fig. 3:**
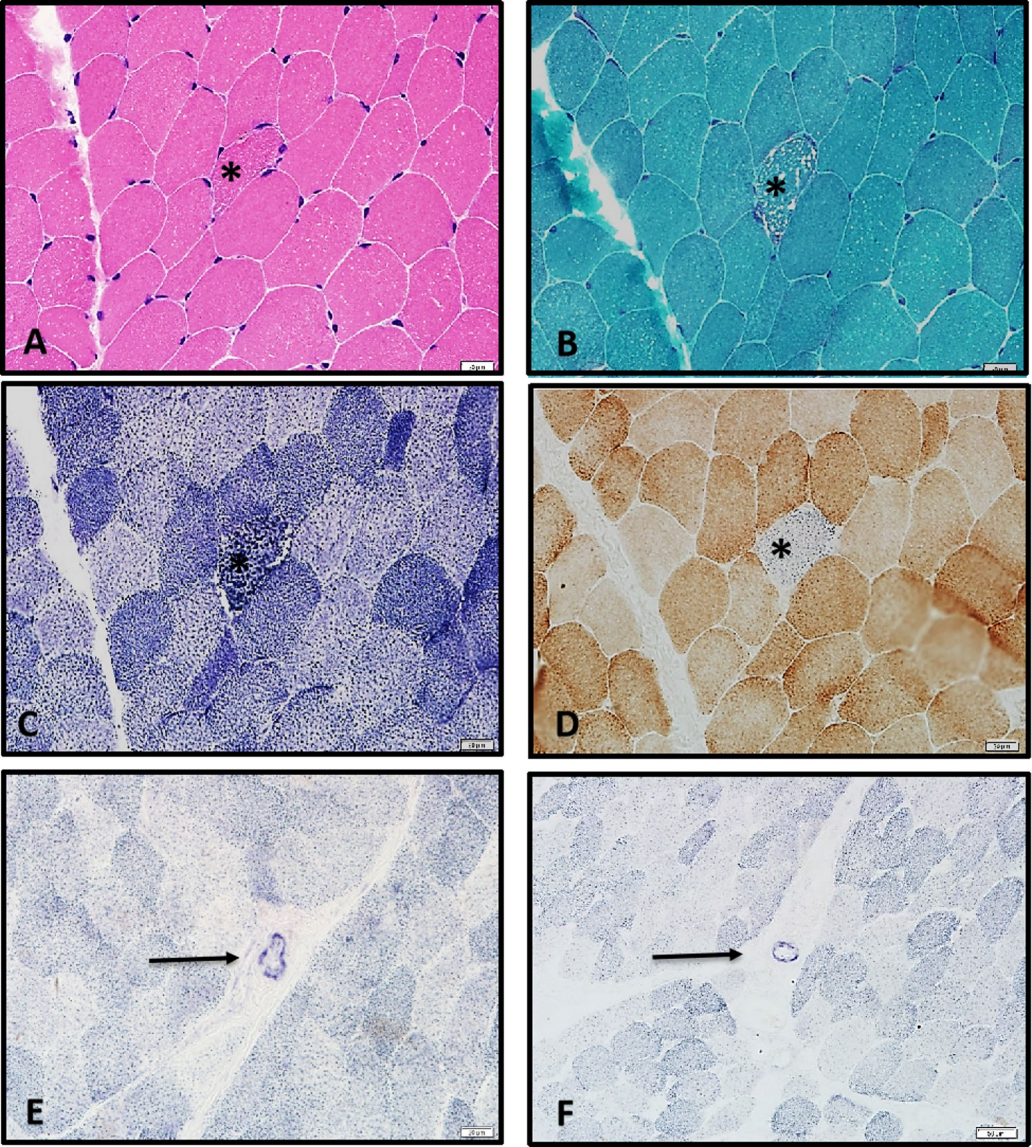
Muscle biopsy of the patient. Muscle biopsy slides (x200). **(A)** H&E staining reveals RRFs. **(B)** MGT staining also reveals RRFs. **(C)** SDH staining reveals that truncated enzyme levels are increased. **(D)** Blue fibers were observed with COX and SDH double staining. **E**,** F.** Enhancement of succinate dehydrogenase reaction (SSV) in blood vessels was observed upon SDH stain

Muscle biopsy showed ragged red fibers (RRFs) on H&E (Fig. 3A) and MGT staining (Fig. 3B). A subset of muscle fibers had COX deficiency, but elevated SDH activity (Fig. 3C). Blue fibers were detected by COX and SDH double staining (Fig. 3D). Additionally, an enhanced succinate dehydrogenase reaction (SSV) was observed in blood vessels on SDH staining (Fig. 3E and F). No significant abnormalities were detected by ATP, PAS, and ORO staining.

### Genomic analysis

**Fig. 4:**
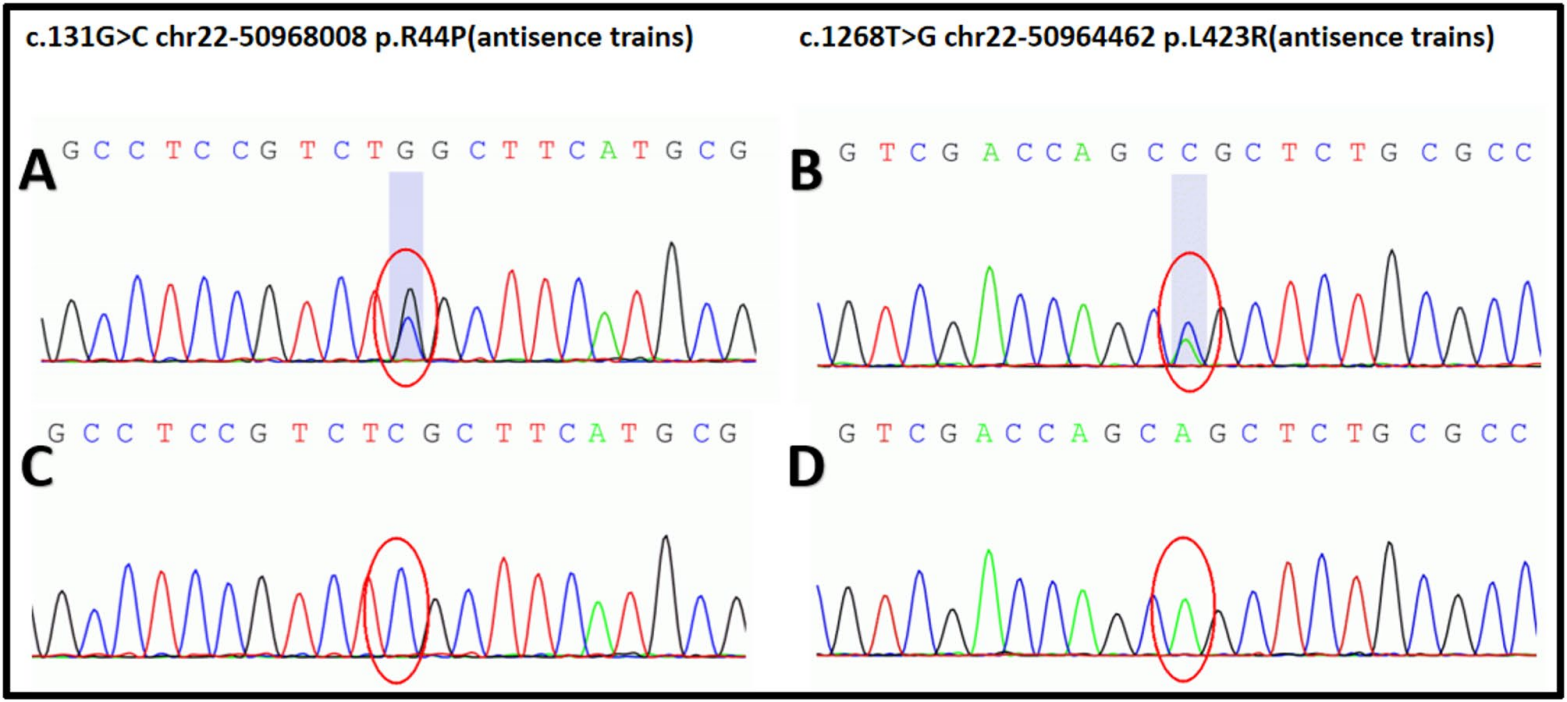
Molecular genetic analysis of the patient. Molecular genetic analysis of the patient identified two heterozygous variants in the *TYMP* gene: c.131G > C (**A**) and c.1268T > G (**B**) in the proband. The wild-type sequences were c.131G (**C**) and c.1268T (**D**)

**Fig. 5:**
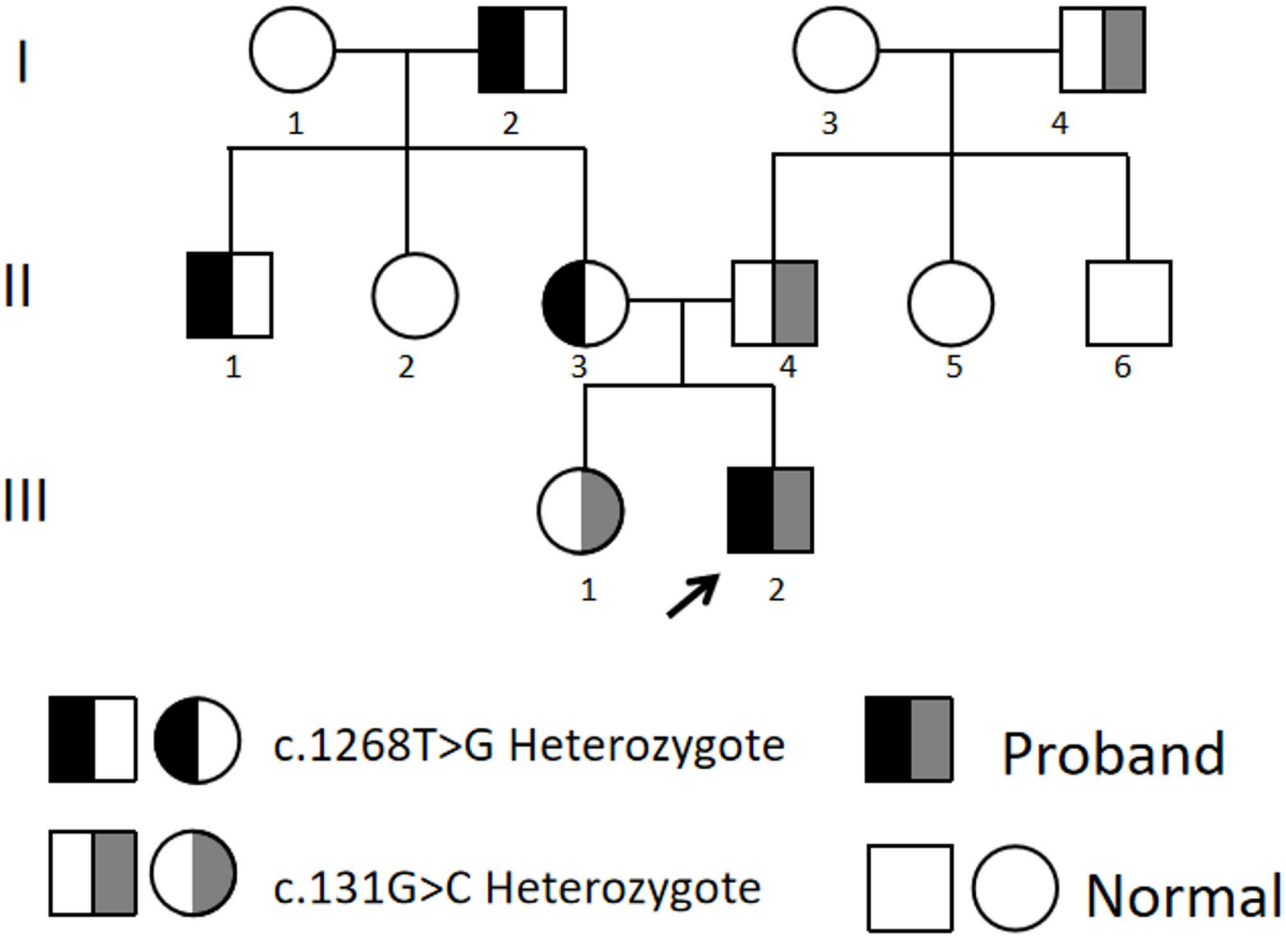
Pedigree of the MNGIE family

Molecular genetic testing of the proband revealed compound heterozygous *TYMP* variants (NM_001953.4: c.1268T > G, and NM_001953.4: c.131G > C). The PCR results are shown in Fig. 4. A T > G substitution at position 1268 caused p.Leu423Arg, while a G > C substitution at position 131 resulted in p.Arg44Pro. As shown in Fig. 5, genetic analysis identified that the proband’s maternal grandfather(I2), uncle(II1), and mother(II3) carried the c.1268T > G heterozygous variant, whereas the proband’s paternal grandfather(I4) faher(II4) and sister(III1) carried the c.131G > C heterozygous variant. All heterozygous carriers have remained asymptomatic to date, consistent with autosomal recessive inheritance. Notably, the c.1268T > G variant represents a novel variant in the *TYMP* gene, reported here for the first time. Mitochondrial DNA sequencing of the patient’s blood detected no pathogenic variants.

### Plasma pyrimidine analysis and TP enzyme analysis

Quantitative serum pyrimidine analysis demonstrated significantly elevated pre-treatment concentrations of thymidine (24.1 µmol/L; normal range < 0.05 µmol/L) and deoxyuridine (11.7 µmol/L; normal range < 0.05 µmol/L). Post-treatment levels remained similarly elevated, with thymidine at 22.4 µmol/L and deoxyuridine at 10.3 µmol/L. Concurrent enzymatic assays demonstrated severely reduced thymidine phosphorylase activity in the patient (23 nmol thymidine formed/hour/mg protein) compared to healthy controls (443.4 ± 79.1 nmol/hour/mg protein; *n* = 10).

### In Silico analysis

**Fig. 6:**
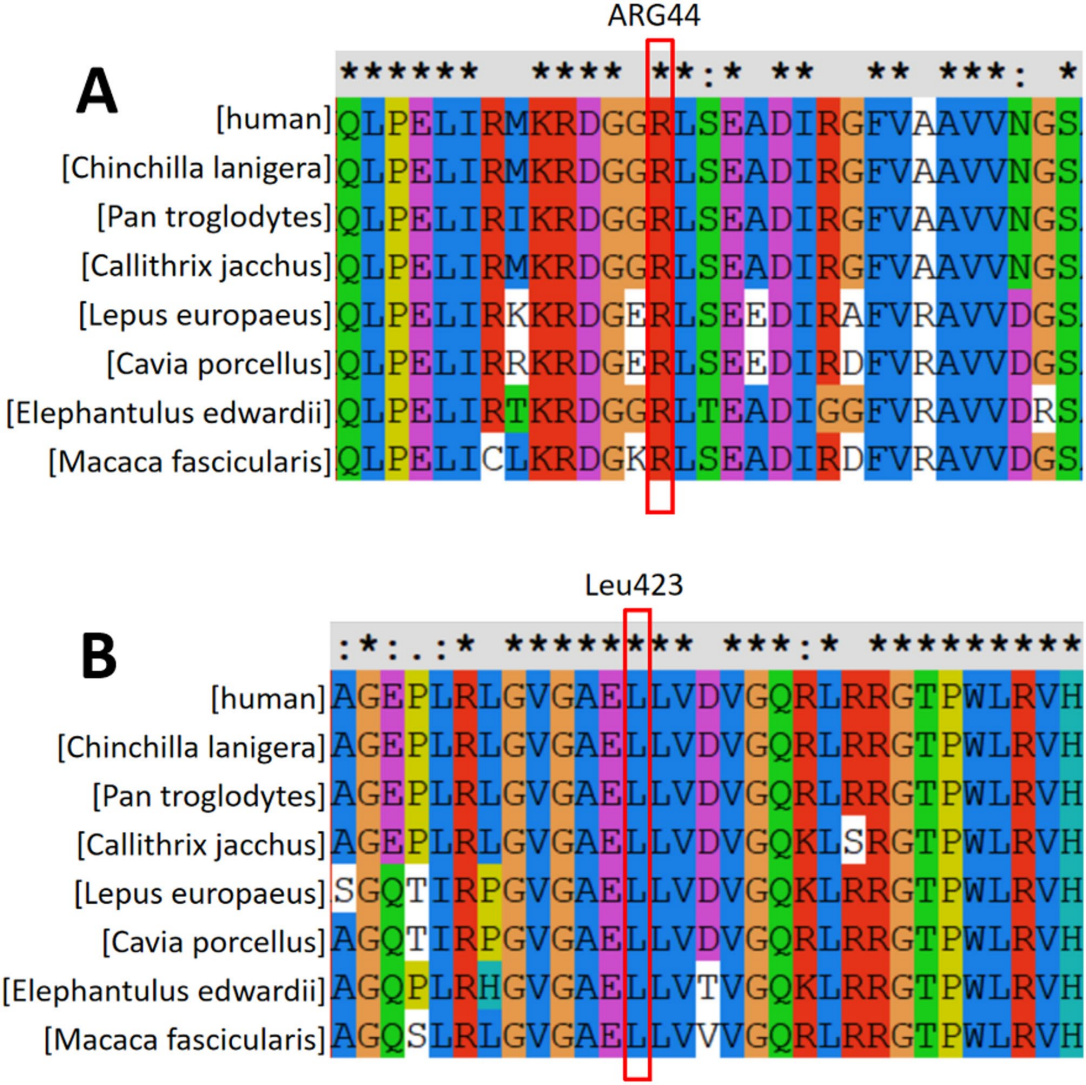
Alignment Results of *TYMP* Gene p.Leu423 in Homologous Species (★, ∶, and · indicate that the amino acid site is completely conserved, highly conserved, and weakly conserved, respectively):

**Fig. 7:**
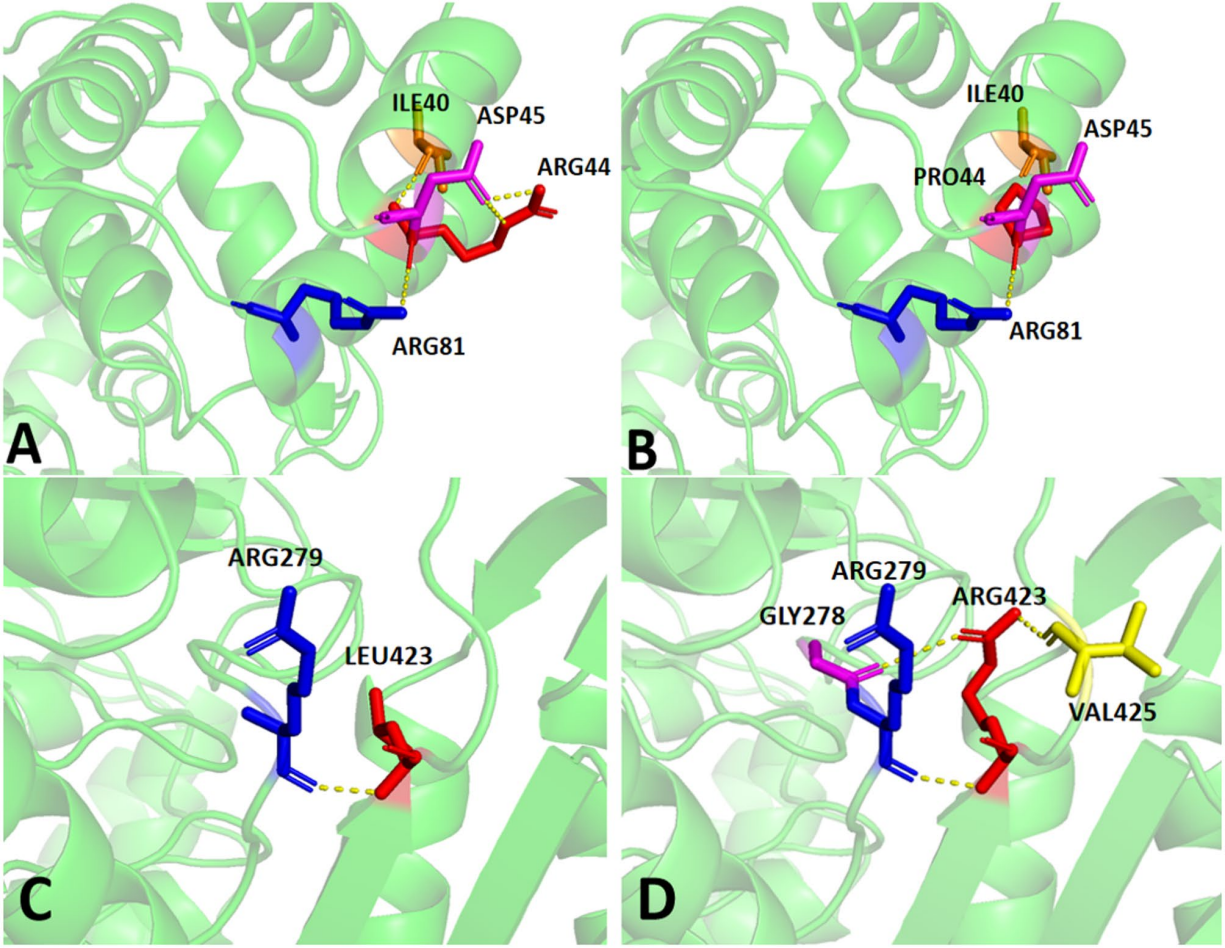
Analysis Diagram of the TP Protein Molecular Model (Yellow dashed lines represent hydrogen bonds) **A**.The p.Arg44 wild-type TP protein molecular model; **B**.The p.Arg44Pro Mutant TP protein molecular model; **C**. The p.Leu423 wild-type TP protein molecular model; **D**. The p.Leu423Arg Mutant TP protein molecular model

Conservation analysis indicated completely conservation of p.Leu423Arg and p.Arg44Pro across eight species (Fig. 6), and both variants were unanimously predicted as likely pathogenic by five online tools (Table 1). Protein modeling of wild-type TP (Arg44) revealed that Arg44 forms a hydrogen bond with Asp45 and two additional bonds with Ile40 and Arg80 (Fig. 7A). These bonds are essential for the hydrophobic core, stabilizing the 3D structure. The missense variant c.131G>C results in Arg44 being replaced by proline (Pro), disrupting hydrogen bonds with Ile40 and Asp45 and causing localized structural changes (Fig. 7B). The p.Leu423Arg variant replaced Leu423 with Arg, enabling Arg to form an additional hydrogen bond with Gly278 (Fig. 7C and D). Gly’s lack of a side chain and high flexibility facilitate this interaction. Arg also formed a hydrogen bond with Val425. As a polar amino acid with a long side chain, Arg exhibits strong hydrogen bonding capability, while Val is non-polar with a short side chain. These new hydrogen bonds may induce local conformational changes, particularly if Val was not previously involved in such interactions.

**Table 1:** Predicted pathogenicity of two variants

Gene Type	Mutation Taster	CADD	PROVEAN	REVEL	SIFT
c.1268T > G (p.Leu423Arg)	1.000	23.9	-4.38	0.9207	0.004
c.131G > C (p.Arg44Pro)	0.983	29.3	-6.28	0.9966	0.014

Mutation taster score results are between 0 and 1, and the larger the value, the more likely it is harmful ; the CADD score threshold is 10–20, and the higher the score, the greater the harm ; the score range of PROVEAN prediction results is-14 ~ 14, the score of-14 ~ 2.5 is ' harmful ‘, and the score of-2.5 ~ 14 is ' neutral ‘. The smaller the score is, the more harmful it is, and vice versa. The REVEL score ranges from 0 to 1, >0.5 was ' harmful ‘, < 0.5 was ' neutral ‘.The SIFT score ranges from 0 to 1, < 0.5 was ' harmful ‘, >0.5 was ' neutral ‘

### ACMG variant classification

According to the ACMG guidelines, the c.131G > A variant can be classified as a likely pathogenic variant (PM3 + PM5 + PP3 + PP4 + PP5). The evidence supporting this classification includes the following: In MNGIE, an autosomal recessive disorder, the c.131G > A variant was confirmed by reverse strand sequencing and parental validation(PM3); The newly identified Arg44Pro missense change affects the same critical residue (Arg44) as the known pathogenic variant Arg44Gln [14](PM5); ClustalX analysis shows complete conservation of Arg44, and predictions from five online tools suggest a potential impact on protein function (PP3); and the proband’s presentation of gastrointestinal complications and neurological deficits is consistent with the clinical features of MNGIE (PP4); literature reports [21, 22] have indicated that this variant is pathogenic(PP5). The c.1268T > G variant can also be classified as a likely pathogenic variant (PM1 + PM2 + PM3 + PP3 + PP4). The variant in TYMP’s exon 9 α/β domain (a pathogenic variant-enriched region containing known functional variants [31]) disrupts TP activity in the absence of benign variation (PM1);The variant is absent from population variant database(PM2); The c.1268T > G variant was confirmed by reverse sequencing and parental testing(PM3); ClustalX analysis shows that Leu423 is completely conserved, and predictions from five online software tools suggest that this variant may impact protein function(PP3); the proband’s presentation of gastrointestinal complications and neurological deficits is consistent with the clinical features of MNGIE (PP4).

### Literature review of Chinese MNGIE cases

Our systematic review identified 15 previously reported cases meeting inclusion criteria. When combined with the current case (Patient 16), this yields a total cohort of 16 patients for analysis. The gastrointestinal and central nervous systems were most frequently affected. Among the patients, 8 manifested initial neurological symptoms, whereas the remaining 8 presented with primary digestive symptoms. Common gastrointestinal manifestations included weight loss/cachexia (100%), diarrhea (62.5%), gastroparesis (43.8%), abdominal pain (31.3%), and vomiting (25%). Neurological manifestations comprised leukoencephalopathy (100%), polyneuropathy (75%), hearing loss (37.5%), ptosis (68.8%), external ophthalmoplegia (62.5%), and vision loss (18.8%). Symptom severity showed heterogeneity, with 2/16 patients (12.5%) exhibiting clinically significant cognitive impairment, while 14 patients maintained normal cognitive function. Biochemical analyses were performed on subsets of the 16-patient cohort, only 5/16 patients underwent thymidine/deoxyuridine testing (all elevated) and 4/16 had TP activity measured (all deficient). The respective tests were not performed in the remaining patients (*n* = 12 nucleoside analysis; *n* = 13 TP activity). The clinical characteristics of patients with MNGIE are shown in Table 2. All patients carried either homozygous or compound heterozygous variants. Missense variants predominated (50%, 11/22), followed by splice donor variants (18.2%, 4/22), frameshift variants (22.7%, 5/22; including duplications and deletions), and nonsense variants (9.1%, 2/22). The detailed variant spectrum is summarized in Table 3.

**Table 2:** Demographic and clinical characteristics of patients with mitochondrial neurogastrointestinal encephalomyopathy

Characteristics Patients (*n* = 16)	
Age of onset, years	(median [IQR]) 19 (17, 26.5)
Age at diagnosis, years	(median [IQR]) 30(20.75, 42.75)
**Gender (n**,** %)**	
Female	8(50)
Male	8(50)
**Phenotypes of MNGIE**	
Early onset	16(100)
late onset	0
**Symptoms and signs (n**,** %)**	
**BMI**	(median [IQR]) 14.54 (13.25, 16.25)
**Neurological symptoms**	
Leukoencephalopathy	16(100)
Peripheral neuropathy	12(75)
Absent refexes	12(75)
Hearing loss	6(37.5)
**Ocular symptoms**	
Ptosis	11(68.75)
External ophthalmoplegia	10(62.50)
Loss of vision	3(18.75)
**Gastrointestinal symptoms**	
Weight loss/cachexia	16(100)
Diarrhea	10(62.50)
Gastroparesis	7(43.75)
Abdominal pain	5(31.25)
Vomiting	4(25)
**Muscle biopsies**	
COX deficient fibers, RRFs, SSVs	5(31.25)
Not done	11(68.75)

BMI: body mass index. RRFs: ragged-red fibers. SSVs: strongly succinate dehydrogenase-reactive blood vessels

**Table 3:** Genetic variants of patients with MNGIE

	TYMP variants	Protein Change	Genotype	Type of variant
P1 [7]	Not Done			
P2 [8]	c.1178_1201dupGGGCGCTGCCGCTGGCGCTGGTGC	p.Arg393_Val400dup	Homo	Duplication
P3 [9]	c.620 C > A	p.Thr207Asn	Comp.Het	Missense
	c.516 + 2T > C	rs797044454		SNP
P4 [10]	c.217G > A	p.Ala73Thr	Homo	Missense
P5 [11]	c.417 + 1G>A	p.?	Homo	SNP
P6 [12]	c.1316G > A	p.Arg439His	Comp.Het	Missense
	c.131G > C	p.Arg44Pro		Missense
P7 [13]	c.776G > A	p.Gly259Glu	Comp.Het	Missense
	c.239_263del	p.Leu80Profs*4		Deletion
P8 [13]	c.776G > A	p.Gly259Glu	Comp.Het	Missense
	c.239_263del	p.Leu80Profs*4		Deletion
P9 [14]	c.914T>C	p.Leu305Pro	Comp.Het	Missense
	c.1319T>C	p.Val440Ala		Missense
P10 [15]	c.131G > C	p.Arg44Pro	Homo	Missense
P11 [16]	c.647-1G > A	rs1295236603	Comp.Het	SNP
	c.597T > G	p.Tyr199*		Nonsense
P12 [17]	c.447dupG	p.His150fs	Homo	Duplication
P13 [18]	c.151G > T	p.Glu51*	Homo	Nonsense
P14 [19]	c.1193-1216dupGGGCGCTGCCGCTGGCGCTGGTGC	p.Arg398_Val405dup	Homo	Duplication
P15 [20]	c.1159 + 1G > A	rs1044840059	Homo	SNP
P16	c.1268T > G	p.Leu423Arg	Comp.Het	Missense
	c.131G > C	p.Arg44Pro		Missense

Het: heterozygote; Comp.Het: compound heterozygote; Homo: homozygous. SNP: single nucleotide polymorphism. Patient 16 is the subject of this report. No corresponding protein modification was identified in the genetic variant of Patient 5. All variants are annotated according to TYMP transcript NM_001953.4

## Discussion

MNGIE, a rare autosomal recessive disorder caused by thymidine phosphorylase deficiency, is frequently diagnosed with significant delay due to its rarity and complex multisystemic manifestations, often requiring multiple specialist referrals over several years before establishing a correct diagnosis [32, 33]. In this study, we describe a severely emaciated male proband with MNGIE carrying compound heterozygous *TYMP* variants: a novel variant (c.1268T > G, p.Leu423Arg) from his asymptomatic mother and a previously reported variant (c.131G > C, p.Arg44Pro) from his asymptomatic father, consistent with autosomal recessive inheritance. The proband developed gastrointestinal symptoms in early childhood, followed by progressive neurological manifestations including paresthesia, numbness, and limb weakness by adolescence (age 17), with subsequent clinical evaluations and diagnostic studies confirming the diagnosis of MNGIE.

Although thymidine phosphorylase is not physiologically expressed in skeletal muscle, muscle tissue from patients with MNGIE exhibits alterations in mtDNA, COX-deficient and ragged red fibers and respiratory chain enzymatic defects [34]. Muscle biopsies from the proband revealed mitochondrial abnormalities, including RRFs, COX-deficient fibres and enhanced SDH activity in blood vessels (SSVs), supporting the MNGIE diagnosis. RRFs and COX deficiency indicate mitochondrial dysfunction, particularly mtDNA abnormalities, central to MNGIE pathogenesis. This finding may explain the leukoencephalopathy observed in MNGIE patients, as mitochondrial dysfunction in blood vessels could disrupt the blood-brain barrier, contributing to neurological manifestations. Vascular mitochondrial dysfunction may play a significant role in the systemic manifestations of MNGIE, including blood-brain barrier disruption leading to leukoencephalopathy or impaired blood flow to the enteric nervous system, which may exacerbate gastrointestinal dysmotility. Although this study did not directly investigate vascular mitochondrial dysfunction, its potential mechanisms warrant further exploration to comprehensively understand the multisystem involvement in MNGIE.

The proband carried compound heterozygous variants ( c.131G>C, p.Arg44Pro and c.1268T>G, p.Leu423Arg) in highly conserved residues, both predicted to be likely pathogenic. Protein modeling analysis revealed that both variants alter the number of hydrogen bonds, thereby impairing the protein’s structural integrity and conformation, ultimately compromising TP function. These findings provide compelling evidence for the functional significance in the pathogenesis of MNGIE. They accentuate the possible role these elements play in impeding the normal activity of thymidine phosphorylase, thereby contributing to the onset and progression of the disease. Consistent with the predominant pattern of homozygous or compound heterozygous missense variants in Chinese MNGIE cases, the proband carried compound heterozygous missense variants. The extreme rarity of MNGIE may result from its recessive inheritance pattern, which requires biallelic variants, combined with diagnostic challenges, thereby hindering the accurate determination of variant prevalence. Notably, the c.131G > C variant identified in our compound heterozygous case has been previously reported in two other Chinese patients, suggesting it may represent a potential mutational hotspot in the Chinese population. Moreover, the same locus (c.131) has been documented to harbor a distinct nucleotide variation (c.131G > A), resulting in an Arg44Gln amino acid substitution [35].

Our literature review revealed an equal gender distribution with no sex-specific phenotypic differences, and 18.75% of cases originated from consanguineous marriages. The disease typically manifested at a median age of 19 years, with diagnostic delay until a median age of 30 years. All patients exhibited the characteristic MNGIE triad (gastrointestinal, neurological, and muscular involvement) with early-onset, rapidly progressive disease and severe cachexia due to chronic malnutrition from gastrointestinal dysmotility. Our case series demonstrates strong concordance with established MNGIE phenotypes across different populations. Consistent with literature reports [2, 36], the cardinal neurological features in our cohort included leukoencephalopathy and peripheral neuropathy, with hearing loss being a common comorbidity. However, we observed some phenotypic variations: (1) ocular manifestations (ptosis and external ophthalmoplegia) showed moderately lower prevalence compared to published rates [36]; and (2) while all patients exhibited cachexia, gastrointestinal symptoms (gastroparesis, abdominal pain, and vomiting) occurred less frequently than previously reported [36]. Additionally, muscle biopsies were performed in five Chinese cases, revealing COX-deficient fibers, RRFs, and SSVs in two patients, while only RRFs were observed in the remaining three. However, the absence of mitochondrial abnormalities in skeletal muscle cannot exclude MNGIE diagnosis, as confirmed cases without muscle pathology have been well documented [37, 38].

Current therapeutic investigations for MNGIE are predominantly focused on two distinct mechanistic approaches. The first strategy involves extracorporeal detoxification through physical removal of accumulated toxic deoxyribonucleosides, primarily utilizing blood purification techniques including hemodialysis and continuous ambulatory peritoneal dialysis (CAPD) [39]. The second therapeutic paradigm aims at enzymatic reconstitution, seeking to restore thymidine phosphorylase activity through various intervention modalities: (i) platelet-derived enzyme replacement via transfusion therapy [32]; (ii) systemic enzymatic correction through allogeneic hematopoietic stem cell transplantation (AHSCT) [8]; (iii) erythrocyte-mediated enzyme delivery using encapsulated thymidine phosphorylase (EE-TP) [40]; and (iv) hepatic enzyme restoration via orthotopic liver transplantation (OLT) [41]. The patient elected to forgo peritoneal dialysis but subsequently showed partial clinical improvement with a combination therapy comprising both targeted nutritional supplementation and mitochondria-specific pharmacotherapy [42].

These findings underscore the multisystemic severity of MNGIE and highlight the critical need for early diagnosis through improved clinical awareness. This is especially important in high-risk populations, such as those with consanguineous family histories or unexplained neurological and gastrointestinal symptoms. Implementing genetic counseling and carrier screening programs could significantly reduce disease incidence by preventing the transmission of biallelic *TYMP* variants. Furthermore, although emerging therapies like gene therapy hold promise, timely diagnostic intervention remains essential for improving outcomes, as delayed management in advanced stages greatly reduces therapeutic efficacy.

## Conclusions

We identified a novel *TYMP* variant (c.1268T > G) and a known variant (c.131G > C) in a Chinese MNGIE patient, expanding the variant spectrum through analysis of 16 cases. The expanded genetic data enhance our understanding of genetic heterogeneity, facilitating accurate diagnosis and targeted therapies. Early genetic screening in high-risk populations, particularly before cachexia and irreversible damage develop, is essential for improving clinical outcomes.

## Data Availability

All data generated or analyzed during this study are included in this published article [and its supplementary information files].

## Abbreviations

MNGIE: mitochondrial neurogastrointestinal encephalopathy
TP: thymidine phosphorylase
*TYMP*: thymidine phosphorylase gene
NGS: genomic analysis involved next-generation sequencing
ACMG: American College of Medical Genetics and Genomics
mtDNA: mitochondrial DNA
HGMD: Human gene mutation database
VUS: Variant of uncertain significance
ERT: enzyme replacement therapy
AHSCT: allogeneic hematopoietic stem cell transplantation
BMI: body mass index
MRI: magnetic resonance imaging
FLAIR: fluid-attenuated inversion recovery
H&E: hematoxylin and eosin
MGT: modified Gomori trichromatic
COX: cytochrome coxidase
SDH: succinate dehydrogenase
NADH: reduced coenzyme 1-like tetrazolium reductase
ATP: adenosine triphosphatase
PAS: periodic acid-Schiff
ORO: oil red-O
CNKI: China National Knowledge Infrastructure
RRFs: ragged red fibers
SSV: succinate dehydrogenase reaction
CAPD: continuous ambulatory peritoneal dialysis
EE-TP: erythrocyte-encapsulated TP
OLT: orthotopic liver transplantation
